# Acadesine suppresses TNF-α induced complement component 3 (C3), in retinal pigment epithelial (RPE) cells

**DOI:** 10.1371/journal.pone.0244307

**Published:** 2020-12-23

**Authors:** Nikolaos E. Efstathiou, Giannis A. Moustafa, Daniel E. Maidana, Eleni K. Konstantinou, Shoji Notomi, Paulo R. T. Barbisan, Constantine D. Georgakopoulos, Joan W. Miller, Demetrios G. Vavvas

**Affiliations:** 1 Angiogenesis laboratory, Department of Ophthalmology, Retina Service, Massachusetts Eye and Ear, Harvard Medical School, Boston, Massachusetts, United States of America; 2 Department of Ophthalmology, University of Patras, Medical School, Patras, Greece; University of Florida, UNITED STATES

## Abstract

**Rationale:**

Age-related macular degeneration (AMD) is the most prevalent form of irreversible blindness in the developed world. Aging, inflammation and complement dysregulation affecting the retinal pigment epithelium (RPE), are considered significant contributors in its pathogenesis and several evidences have linked tumor necrosis factor alpha (TNF-α) and complement component 3 (C3) with AMD. Acadesine, an analog of AMP and an AMP-activated protein kinase (AMPK) activator, has been shown to have cytoprotective effects in human clinical trials as well as having anti-inflammatory and anti-vascular exudative effects in animals. The purpose of this study was to evaluate if acadesine is able to suppress TNF-α induced C3 in RPE cells.

**Methods:**

ARPE-19 and human primary RPE cells were cultured and allowed to grow to confluence. TNF-α was used for C3 induction in the presence or absence of acadesine. Small molecule inhibitors and siRNA were used to determine if acadesine exerts its effect via the extracellular or intracellular pathway and to evaluate the importance of AMPK for these effects. The expression level of C3 was determined by immunoblot analysis.

**Results:**

Acadesine suppresses TNF-α induced C3 in a dose dependent manner. When we utilized the adenosine receptor inhibitor dipyridamole (DPY) along with acadesine, acadesine’s effects were abolished, indicating the necessity of acadesine to enter the cell in order to exert it’s action. However, pretreatment with 5-iodotubericidin (5-Iodo), an adenosine kinase (AK) inhibitor, didn’t prevent acadesine from decreasing TNF-α induced C3 expression suggesting that acadesine does not exert its effect through AMP conversion and subsequent activation of AMPK. Consistent with this, knockdown of AMPK α catalytic subunit did not affect the inhibitory effect of acadesine on TNF-α upregulation of C3.

**Conclusions:**

Our results suggest that acadesine suppresses TNF-α induced C3, likely through an AMPK-independent pathway, and could have potential use in complement over activation diseases.

## Introduction

Age-related macular degeneration (AMD), is a vision threatening progressive retinal disease and the primary leading cause of vision loss in the western world [1]. Its prevalence follows an escalating pattern, and projection on the disease’s burden approximates a number of 288 million people to be affected in the next 20 years [1]. AMD manifests in two major forms. The non-neovascular, non-exudative “dry” form, which is the most prevalent one, accounting for 85–90% of patients affected and the neovascular or exudative form affecting 10–15% of patients [2]. Dry AMD is mainly characterized by accumulation of deposits (drusen) [3–5] under the retinal pigment epithelium (RPE) [4, 6] and neurosensory retina, accompanied by degeneration of RPE and neurosensory retina. Neovascular AMD is mainly characterized by the development of choroidal neovascularization accompanied by leakage of fluid, lipid deposition, hemorrhages and fibrotic scaring [2, 7]. Although the discovery of anti-vascular endothelial growth factor (anti-VEGF) therapies has led to effective treatment of wet AMD [8] no effective treatment is available for the dry form.

Multiple biological pathways are involved in AMD pathogenesis [7, 9–11] with complement system in particular to play a seminal role. Genetic studies revealed that gene polymorphisms in complement factor H (CFH), a regulator of alternative complement pathway, significantly increases the odds of AMD and may affect progress in response to treatment [12–16]. Other studies uncovered the association of additional complement system genes with AMD including CFB/C2 [17, 18], CFI [19, 20], CFD [21] and C3 [22, 23].

(C3) is involved in all three complement pathways. The pivotal step in all of them is the conversion of C3 to C3b. C3 consists of C3 alpha chain and C3 beta chain linked with disulfide bonds. Activation occurs after the convertase mediated cleavage of C3, that is the first step among many, to generate up to 12 C3 cleavage products [24–26]. Even though the exact mechanism of complement system contribution to AMD is not fully understood there is significant amount of evidence linking C3 and its cleavage products with AMD, including genetic evidence [22, 23], elevated plasma levels [27, 28] and histological data [29, 30]. Direct inhibition of C3 has been tested in a phase 2 clinical trial with potential positive signal in slowing down progression of the disease [31]. However, direct inhibition of C3 was also accompanied with increased conversion to exudative AMD [31].

Acadesine (5-Aminoimidazole-4-carboxamide-1-β-D-ribofuranoside) is an adenosine analog which has cytoprotective properties [32] as well as anti-inflammatory and anti-exudative properties [33–37]. Acadesine is taken up into cells by adenosine transporters [38] and subsequently is phosphorylated by adenosine kinase to generate ZMP, an adenosine monophosphate (AMP)-mimetic and activator of AMPK [39]. Activation of AMPK has been shown to suppress inflammation, and ameliorate exudation [33–37, 40–46] and has been implicated in vascular cytoprotection against complement-mediated injury [47]. Hence, we wanted to explore the potential effect of acadesine in suppressing inflammatory induction of C3 in RPE cells and explore its mechanism of action.

## Results

### Acadesine inhibits TNF-α induced C3 expression in RPE cells

To investigate if acadesine inhibits inflammatory C3 induction we examined two different human RPE cell lines and employed TNF-α which has been shown to induce C3 expression [48, 49]. In particular, 24 hours after serum depletion, ΑRPE-19 and human fetal primary epithelial cells (hfRPE) were treated with various dosages of acadesine (0.25-2mM) starting 1 hour before treatment with TNF-α (10ng/ml, 24 h) and the expression levels of C3 protein were assessed by western blotting.

**Fig 1:**
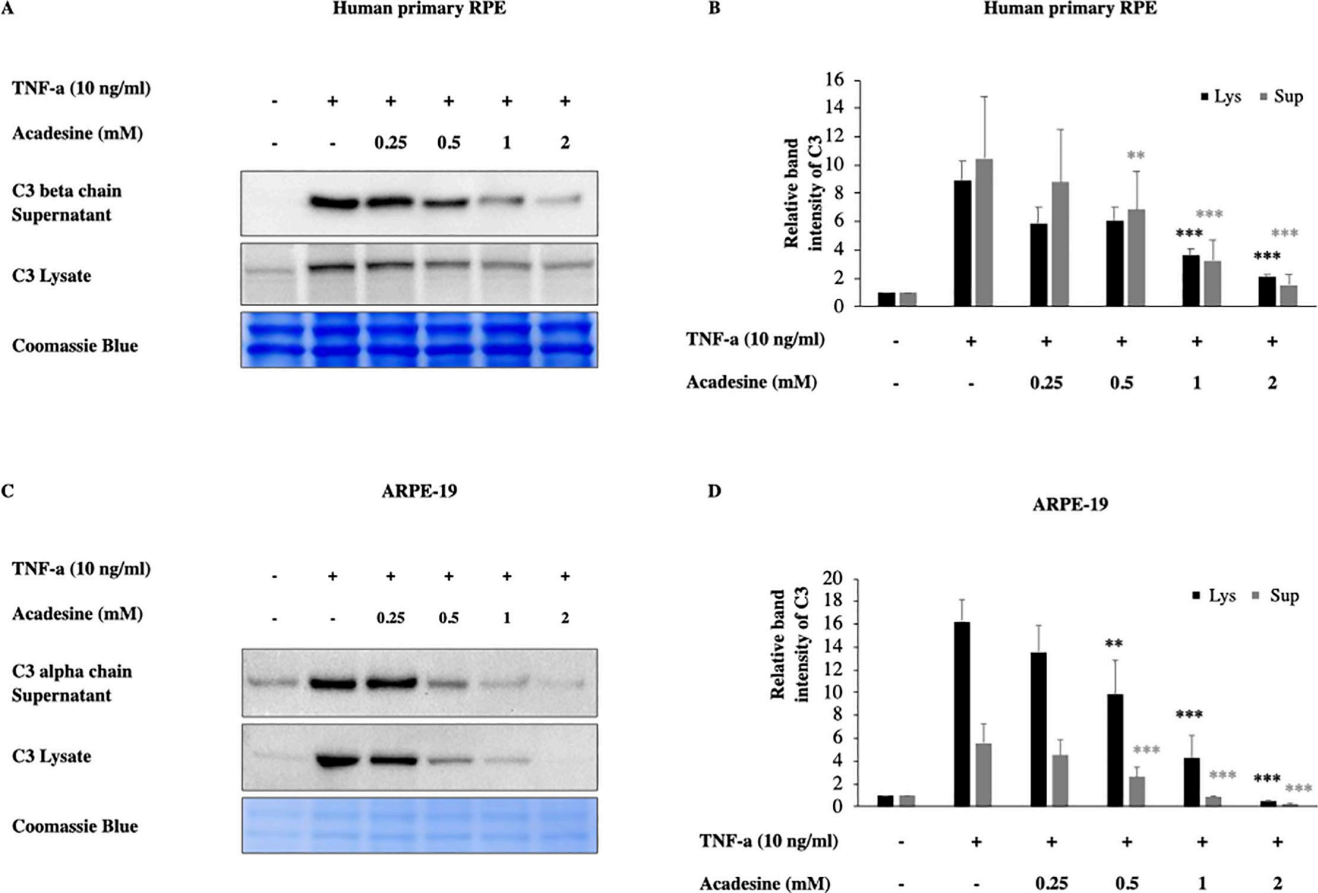
Acadesine inhibits TNF-α induced C3 in RPE cells. Following 24h starvation by serum depletion RPE cells were pretreated by various dosages of acadesine (0.25-2mM) for 1 hour before stimulation with TNF-α (10ng/ml) for another 24 hours. **A.** Western blot representing the expression levels of C3 in human primary RPE cell lysates and supernatants. **B.** Densitometry analysis of C3 levels in human primary RPE cell lysates and supernatants. **C.** Western blot representing the expression levels of C3 in ARPE-19 cell lysates and supernatants. **D.** Densitometry analysis of C3 levels in ARPE-19 cell lysates and supernatants. Each group represents the mean of at least three independent experiments. *p<0.05, **p<0.01, ***p<0.001 compared with the TNF-α group. Coomassie blue indicates the relative loading of the samples.

Acadesine treatment of ARPE-19 and hfRPE cells abrogated TNF-α induced C3 expression levels in a dose dependent manner. In hfRPE cells treatment with acadesine 0.25mM and 0.5mM had similar effects and caused a reduction of ~32% (mean±SE 32.39±8.73) and (31.95±1.24) of TNF-a induced C3 expression. Treatment with acadesine 1mM reduced C3 levels by ~57% (56.7±7.17) while the highest dose of acadesine resulted in ~74% (73.52±6.35) reduction of TNF-a induced C3 expression levels. In ARPE-19 cells treatment with acadesine 0.25mM led to ~17% (17.26±4.17) reduction while 0.5mM of acadesine resulted to ~41% (40.68±12.75) reduction. Treatment with higher dose of acadesine 1mM reduced TNF-a induced C3 expression levels by ~76% (75.74±8.31) while treatment with the highest dose of acadesine 2mM suppressed TNF-a induced C3 levels by ~97% (97.13±1.1) (Fig 1 and S1 Fig). Acadesine suppression of full length cytosolic C3 led to corresponding reduction in cleaved products detected in the cultured media as well (Fig 1 and S1 Fig).

### Dipyridamole (DPY) abolishes the inhibitory action of acadesine on TNF-α induced C3 expression

**Fig 2:**
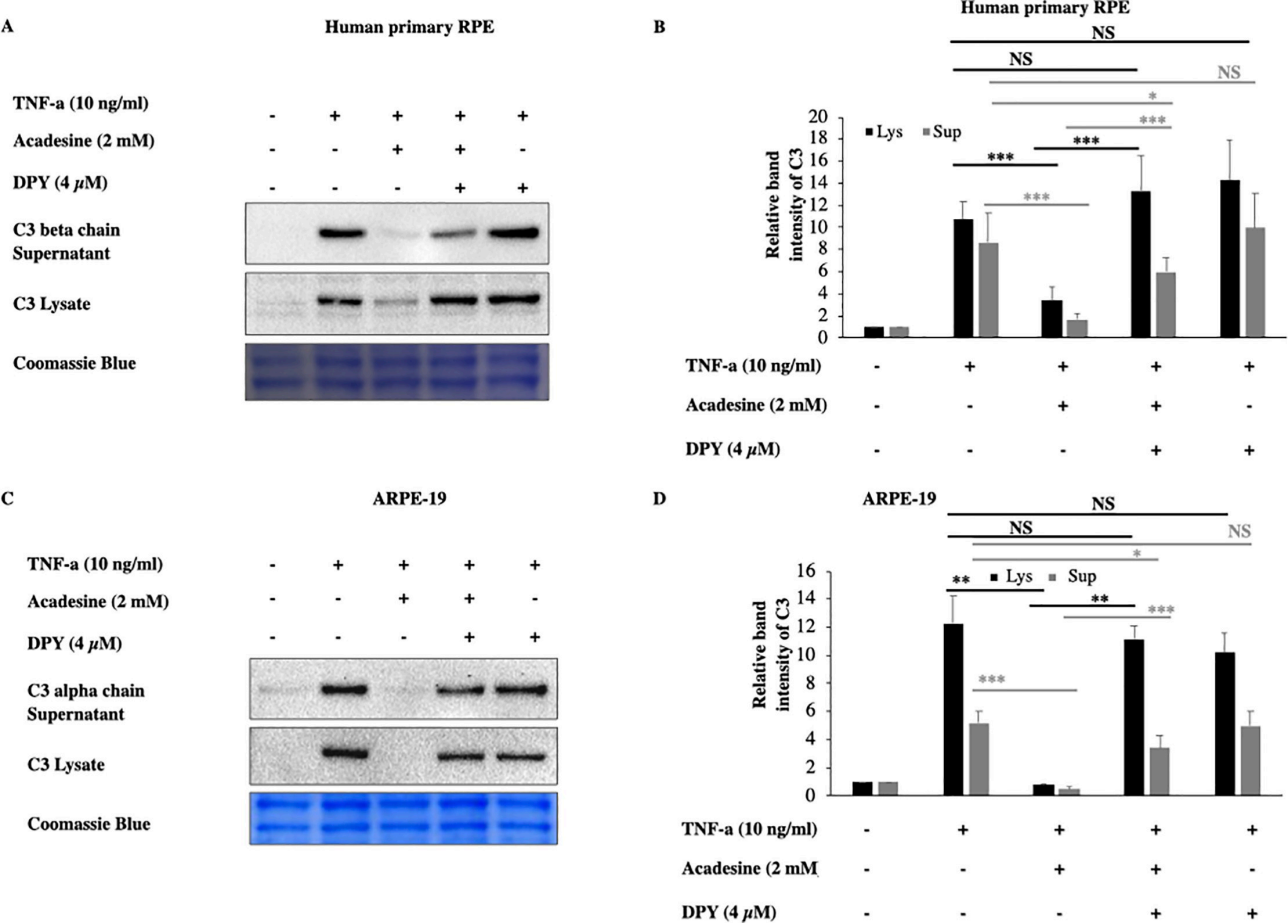
Dipyridamole abolishes the inhibitory effects of acadesine on TNF-α induced C3 expression. Following 24 hours starvation by serum depletion RPE cells were pretreated with dipyridamole (4μM) for 1 hour and then treated with acadesine (2mM) for 1 hour before stimulation with TNF-α (10ng/ml) for additional 24 hours. Pretreatment with dipyridamole prevented acadesine from exerting its inhibitory effects as seen in **A.** Western blot representing the expression levels of C3 in human primary RPE cell lysates and supernatants. **B.** Densitometry analysis of C3 levels in human primary RPE cell lysates and supernatants. **C.** Western blot representing the expression levels of C3 in ARPE-19 cell lysates and supernatants. **D.** Densitometry analysis of C3 levels in ARPE-19 cell lysates and supernatants. Each group represents the mean of at least three independent experiments. *p<0.05, **p<0.01, ***p<0.001. Coomassie blue indicates the relative loading of the samples.

Acadesine, can exert its effects through either extracellular or intracellular mechanisms. To examine if acadesine’s effects are mediated via an extracellular or via an intracellular mechanism we used dipyridamole (DPY) which blocks the cell membrane adenosine transporters and prevents acadesine uptake into the cells [37]. Pretreatment with 4 μM of DPY abolished the effect of acadesine on TNF-α induced C3 protein expression in both human primary and ARPE-19 cell lysates (Fig 2). These findings suggest that acadesine uptake into the cells is necessary in order to suppress TNF-α induced C3 expression.

### Activation of AMPK by acadesine is not needed for the suppressive action of acadesine on TNF-α induced C3 expression

**Fig 3:**
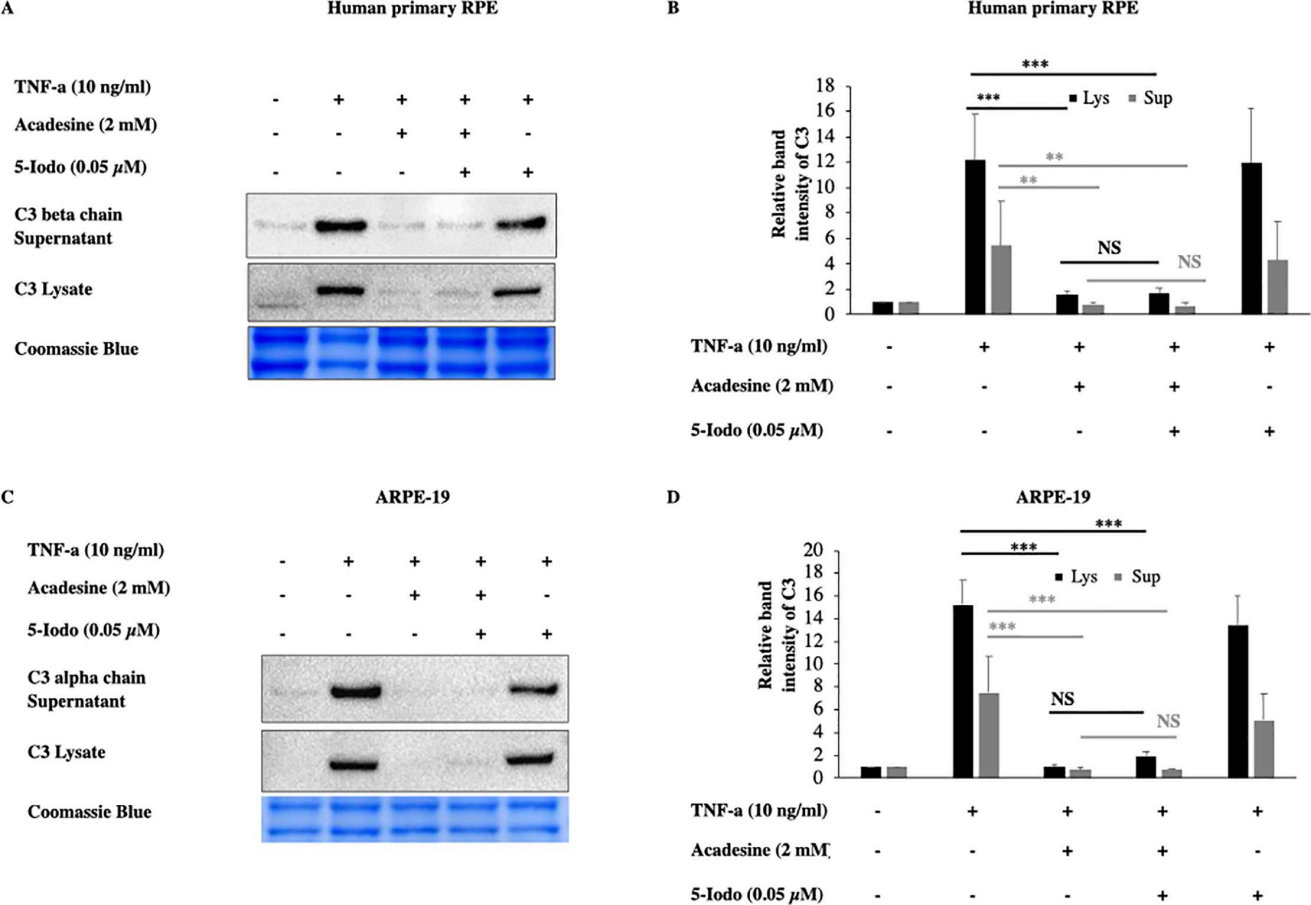
The inhibitory effects of acadesine on TNF-α induced expression of C3 were not affected by 5-Iodotubericidine. Following 24 hours starvation by serum depletion RPE cells were pretreated with 5-iodotubericidine (0.05μM) for 1 hour and then treated with acadesine (2mM) for 1 hour before stimulation with TNF-α (10ng/ml) for additional 24 hours. Pretreatment with 5-iodotubericidin did not prevent acadesine from exerting its suppressive effects as seen in **A.** Western blot representing the expression levels of C3 in human primary RPE cell lysates and supernatants. **B.** Densitometry analysis of C3 levels in human primary RPE cell lysates and supernatants. **C.** Western blot representing the expression levels of C3 in ARPE-19 cell lysates and supernatants. **D.** Densitometry analysis of C3 levels in ARPE-19 cell lysates and supernatants. Each group represents the mean of at least three independent experiments. *p<0.05, **p<0.01, ***p<0.001. Coomassie blue indicates the relative loading of the samples.

Once acadesine is taken up by the cells, it can be phosphorylated by AK into its monophosphorylated form (ZMP) which consequently can activate AMPK. To examine if this is the mechanism by which acadesine suppresses C3, we used the adenosine kinase inhibitor 5-Iodotubericidin (5-Iodo). Application of 5-Iodo at a dose (0.05μM) was able to prevent AMPK activation by acadesine (S5 Fig) but did not prevent the suppressive effect of acadesine on TNF-α induced C3 expression in both human primary RPE and ARPE-19 cells (Fig 3). These findings suggest that acadesine conversion to ZMP (activator of AMPK) is dispensable for its suppressive action on TNF-α induced C3 expression.

**Fig 4:**
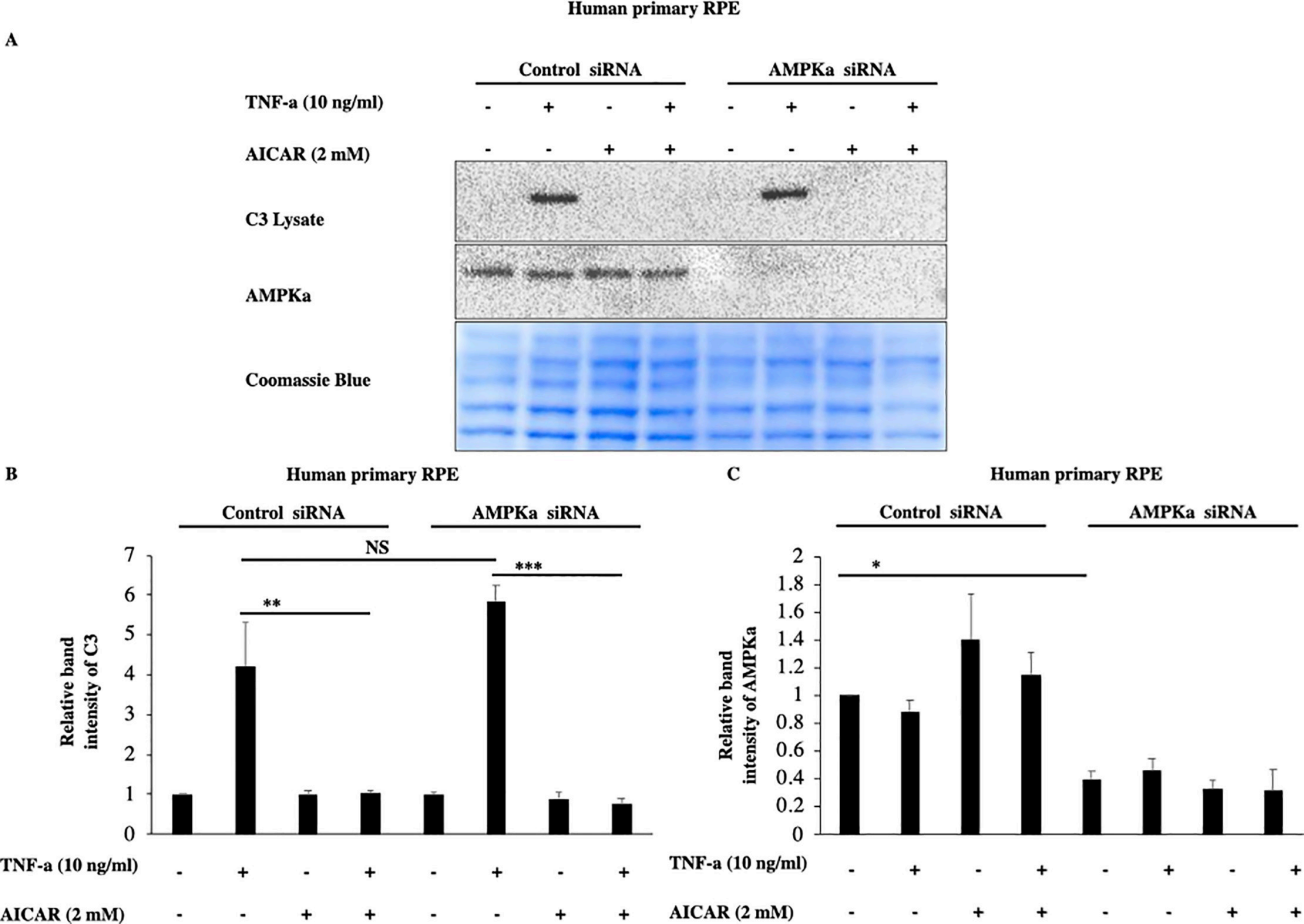
Silencing of AMPKα expression by siRNA in RPE cells did not affect the inhibitory effects of Acadesine on TNF-α induced C3 expression. The third day after transfection with either non-targeting control siRNA or AMPKα1 and AMPKα2 siRNA medium was replaced with fresh serum free medium. 24 hours after serum depletion pRPE cells were treated with acadesine (2mM) for 1 hour before stimulation with TNF-α (10ng/ml) for additional 24 hours. TNF-α induced C3 levels were similar in both groups. AMPKα knockdown did not abolish the inhibitory effect of acadesine in TNF-α induced C3 expression as seen in **A.** Western blot representing the expression levels of C3 in transfected human primary RPE cell lysates. **B.** Densitometry analysis of C3 protein levels in transfected human primary RPE cells. **C.** Densitometry analysis of AMPKα levels in human primary RPE cells showing significant reduction in protein expression in cells transfected with AMPKα1+ AMPKα2 siRNA compared with those transfected with non-targeting control siRNA.

To acquire further evidence that these effects of acadesine are not mediated by AMPK activation we used siRNA to knock down the expression of both AMPK alpha catalytic isoforms (α1 and α2). TNF-α induced C3 expression levels were similar in both scrambled and AMPKα specific siRNA treated groups (Fig 4). Furthermore, AMPKα downregulation didn’t modify acadesine’s effect on TNF-α induced C3 in human primary RPE cells (Fig 4). These results suggest that AMPKα catalytic subunit is likely dispensable for acadesine’s effect on TNF-α induced C3 and hence indicate that acadesine’s inhibitory action on TNF-α induced C3 is largely AMPK independent at least in RPE cells.

## Discussion

C3 is the most abundant complement component, is involved in all three complement cascade pathways and certain single nucleotide polymorphisms are associated with higher risk of developing AMD [22, 23, 50–59]. Furthermore, elevated plasma levels of C3 have been reported in AMD patients [27, 28, 60, 61]. Measurement of plasma levels of C3d and evaluation of C3d/C3 ratio directly [28, 62] or in correlation with known risk CFH variants [63], indicated increased systemic complement activation in AMD patients and provided further evidence of the connection between C3 and AMD.

Although C3 is mainly synthetized by the liver, detection of C3 mRNA has been reported in retina, choroid and RPE cells [22, 49, 64]. Antibodies against C3 and C3 fragments showed various degrees of immunoreactivity in drusen deposits [30, 64, 65] and in infiltrating immune cells [66]. Correlation of C3 with AMD has led to clinical trial of C3 inhibitor in geographic atrophy patients, with reported positive results, however, a significant number of patients (~20%) converted to exudative AMD [31]. This increase in exudation is in line with a reliability animal study that reported increased exudation in animal models of CNVM [67].

TNF-α has been implicated in AMD [68, 69], can upregulate C3 and can itself be upregulated by C3 as well [48, 70]. In this study we used TNF-α to induce complement activation in vitro and we observed increased C3 expression in RPE cells 24 hours after TNF-α treatment. This result is in agreement with previous studies in which inflammatory cytokines like TNF-α induced complement component synthesis [48, 49]. Acadesine has anti-inflammatory properties [33, 34, 37, 45], is an exercise mimetic [71, 72] and has been used in human clinical trials as a cardioprotectant [73, 74]. Pre-treatment of human RPE cells with the AMP analog, acadesine, downregulated TNF-α induced C3 and its cleaved products C3α and C3β.Cleavage of C3 alpha chain leads to C3a, which is an anaphylatoxin and is implicated in a variety of studies with AMD pathology. Furthermore it has been suggested that C3a is sufficient to lead in decreased proteasome-mediated proteolytic activity on human RPE cells and in a mouse model.

The observed acadesine effects were not a result of extracellular actions since using DPY, a molecule that prohibits the entrance of acadesine into the cells [37], abolished the suppressing action of acadesine on TNF-α induced C3, suggesting that acadesine exerts its effects via the intracellular pathway. Since AK can phosphorylate acadesine and convert it to the AMPK activator ZMP we examined if AMPK is important to mediate the observed effect of acadesine. Using the AK inhibitor 5-Iodo to prevent AMPK activation [75] by acadesine (S5 Fig), we found that acadesine could still exert its suppressive action on TNF-α induced C3, suggesting that its mechanism of action does not depend on AMPK. This was further verified by the negligible effect on acadesine’s inhibitory action after siRNA silencing of AMPKα expression. These findings indicate that the effect of acadesine on TNF-α induced C3 is likely AMPK independent. Although many times acadesine exerts its effects through AMPK, there are several reports that have found that acadesine was able to inhibit inflammation in an AMPK independent manner [76–80]. However, the exact molecular mechanism remains obscure.

The strong association between complement dysregulation and AMD highlights the significance of finding therapeutics that can regulate complement overactivation. Existing data from clinical trials show that complete inhibition of complement system may not be the most beneficial approach [31, 81–83]. Perhaps agents that prevent the overactivation rather than agents that inhibit complement completely may be a more optimal approach. Here, we provide evidence that the small molecule acadesine could diminish TNF-α induced C3, C3α and C3β to levels similar to baseline in an in vitro system. Acadesine may be considered as a regulator of C3, in complement overactivation conditions. Further long term animal studies are needed to gather important information for optimal use.

## Methods

### Reagents

Acadesine was purchased from Toronto Research Chemicals (Toronto, ON, Canada). Tumor Necrosis Factor a (TNF-α, 210-TA) was acquired from R&D Systems (Minneapolis, MN, USA). 5-iodotubericidin (5-Iodo, I100) and Dipyridamole (DPY, D9766) were purchased from Millipore SIGMA (St. Louis, MO, USA). C3 (sc-28294) antibody that was used in human primary RPE cells and C3 (sc-52629) antibody that was used in ARPE-19 cells were from Santa Cruz Biotechnology (Dallas, Tx, USA). Full list of the antibodies and dilutions used is provided in S1 Table.

### Cell culture procedures

ARPE-19 cells (CRL-2302) were purchased from ATCC (Manassas, VA, USA) and primary human fetal retinal pigment epithelial cells, H-RPE (00194987) were from Lonza (Basel, Switzerland). DMEM:F12 (11320–033) was purchased from Thermo Fisher Scientific (Waltham, MA, USA). RtEGM BulletKit (00195409) which is a culture system containing RtEBM Basal medium (00195406) and RtEGM Single Quots Supplements (00195407) was purchased from Lonza. ARPE-19 cells were cultured in DMEM:F12 medium containing 100 U/ml penicillin and 100ug/ml streptomycin supplemented with 10% FBS. H-RPE cells were cultured in RtEBM containing Single Quots supplements with the addition of 2% FBS for the first 24hrs or serum free thereafter. Cells were cultured as described until they reach confluent state. Upon confluence, serum was depleted for 24 hours and cells were treated appropriately for another 24 hours. Acadesine was added 1hour before TNF-α and 5-IODO or DPY were added 1 hour prior to Acadesine. Experiments were performed on ARPE-19 at passage 6–9 and on H-RPE at passage 3–4.

### Protein extraction

Following treatment culture medium was collected and cleared by centrifugation (14.000g x 15 minutes, 4 ^o^C). Then supernatants were collected and used for western blotting or stored at -80 ^o^C. Cells were lysed on ice with lysis buffer [20mM NaHEPES, 20mM KCL, 20mM NaF, 20mM b-glycerophosphate, 20mM Sodium pyrophosphate, 2.5mM EGTA, 2.5mM EDTA, 1%Triton X-100, 0.1% 2-mercaptoethanol and protease inhibitor cocktail (ROCHE, 11836170001)] [84]. Lysates were cleared by centrifugation (14.000g x 15 minutes, 4^o^c) and total sample protein was measured by Bradford assay (Thermo Fisher Scientific, 23236) before samples were used for western blotting or stored in -80 ^o^C.

### Western blotting

For western blotting samples (15 micrograms of total protein) were reduced in SDS sample buffer containing 10% 2-mercaptoethanol at 90 ^0^C for 10 min and then loaded for electrophoresis in 4–12% Bis-Tris Polyacrylamide gels [85]. Proteins were transferred on a 0.45μm PVDF membrane at 25V at 4 ^0^C overnight. Successful transfer and equal protein loading were verified by Brilliant Blue staining (Sigma, B2025) (0.1%brilliant blue in 50% methanol, 40% H_2_O and 10% acetic acid) [86]. Membranes were de-stained by washing with washing solution (70% methanol, 20% H_2_O, 10% acetic acid and 0.1% 10N NaOH). Membranes were blocked with blocking solution [5% non-fat dry milk in TBST (0.05% Tween-20)] for 25 minutes at room temperature and then incubated with primary antibodies for 3hours at room temperature. Washed 4 times for 3 minutes each in TBST and then incubated with secondary HRP-conjugated antibodies accordingly for 25 minutes at room temperature before final washing step, 4 times for 3 minutes each. Membranes were developed using chemiluminescent substrate (ECL Select western blotting detection reagent, RPN 2235, GE Healthcare Life Sciences, USA) [87] and images captured by using the ChemiDoc imaging system (Bio-Rad, Hercules, CA, USA). For full list of the antibodies used see S1 Table.

### Silencing of AMPKα expression by siRNA

Human primary RPE cells were plated in 12-well plates and transfected with ON-TARGETplus Human PRKAA1 siRNA- SMARTpool, (L-005027-00-0005) and ON-TARGETplus Human PRKAA2 siRNA- SMARTpool, (L-005361-00-0005) or ON-TARGETplus Non-targeting control siRNA, (D-001810-01-05) from Dharmacon (Lafayette,CO,USA) utilizing Lipofectamine RNAiMAX (Invitrogen by Thermo Fisher Scientific,13778) according to the manufacturers protocol. Medium was changed after 24hours. On day 3 medium changed with fresh serum free medium for 24 hours and then treatment with acadesine (2mM) and/or TNF-α (10ng/ml) followed accordingly for another 24 hours. Then culture supernatants collected and lysates processed as previously.

### Quantification and statistical analysis

All experiments were performed three times and data are presented as the mean of these three independent experiments. Quantification of blots performed by using the program ImageJ (Fiji). For data analysis we used Anova and Tukey test was used for multiple comparisons. The analysis was performed with the statistical package Stata 15. Differences considered significant when at least p<0.05. *p<0.05, **p<0.01, ***p<0.001. Error bars in the graphs represent the standard error of mean.

## Supporting information

S1 Fig **A.** Extended frame of the same blot of ARPE-19 lysates in Fig 1C. The antibody used for C3 detection also detected a band of lower molecular weight at around 110kD, that was corresponding to C3 alpha chain. **B.** Extended frame after overexposure of the same blot of Human RPE supernatants in Fig 1A. The C3 beta chain bands are saturated. At the upper part of the blot it is detected weak signal of C3. The C3 bands follow the same diminishing trend as in lysates where the C3 signal is much stronger. **C.** Extended frame after overexposure of the same blot of ARPE19 supernatants in Fig 1C. The C3 alpha chain bands are saturated. At a higher molecular weight it is detected weak signal of C3. The C3 bands follow the same inhibitory trend as in lysates where the C3 signal is much stronger.
(TIFF)

S2 Fig **A.** Whole blot image of the Coomassie staining corresponding to Fig 1A. **B.** Whole blot image of the Coomassie staining corresponding to Fig 1C.
(TIFF)

S3 Fig **A.** Whole blot image of the Coomassie staining corresponding to Fig 2A. **B.** Whole blot image of the Coomassie staining corresponding to Fig 2C.
(TIFF)

S4 Fig **A.** Whole blot image of the Coomassie staining corresponding to Fig 3A. **B.** Whole blot image of the Coomassie staining corresponding to Fig 3C.
(TIFF)

S5 Fig Acadesine induced AMPK activation (P-ACC) in the presence of TNF-a. Blockage of conversion of acadesine to ZMP by employment of 5-Iodo (0.05μM) prevented the AMPK activation (P-ACC) as seen in **A.** Western blot representing the AMPK activation (P-ACC) in human primary RPE lysates. **B.** Western blot representing the AMPK activation (P-ACC) in ARPE-19 cells lysates.
(TIFF)

S6 Fig **A.** Whole blot image of the Coomassie staining corresponding to S5A Fig. **B.** Whole blot image of the Coomassie staining corresponding to S5B Fig.
(TIFF)

S7 Fig **A.** Whole blot image of the Coomassie staining corresponding to Fig 4A.
(TIFF)

S1 Table List of the antibodies and dilutions used. (TIFF)
