# Supplementary figures and images for: Acadesine suppresses TNF-α induced complement component 3 (C3), in retinal pigment epithelial (RPE) cells
Source: PLoS One. 2020 Dec 23;15(12):e0244307. doi: 10.1371/journal.pone.0244307 (PMC7757886; doi:10.1371/journal.pone.0244307)

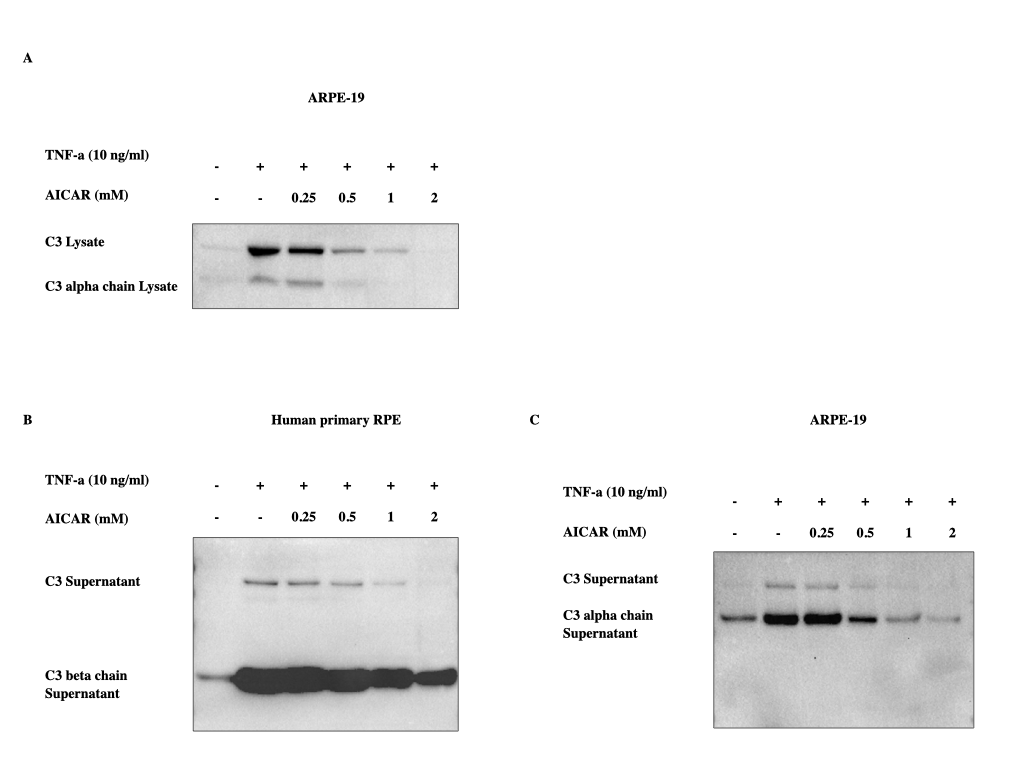

Supplement: S1 Fig — A. Extended frame of the same blot of ARPE-19 lysates in Fig 1C. The antibody used for C3 detection also detected a band of lower molecular weight at around 110kD, that was corresponding to C3 alpha chain. B. Extended frame after overexposure of the same blot of Human RPE supernatants in Fig 1A. The C3 beta chain bands are saturated. At the upper part of the blot it is detected weak signal of C3. The C3 bands follow the same diminishing trend as in lysates where the C3 signal is much stronger. C. Extended frame after overexposure of the same blot of ARPE19 supernatants in Fig 1C. The C3 alpha chain bands are saturated. At a higher molecular weight it is detected weak signal of C3. The C3 bands follow the same inhibitory trend as in lysates where the C3 signal is much stronger. (TIFF) [file pone.0244307.s001.tiff]

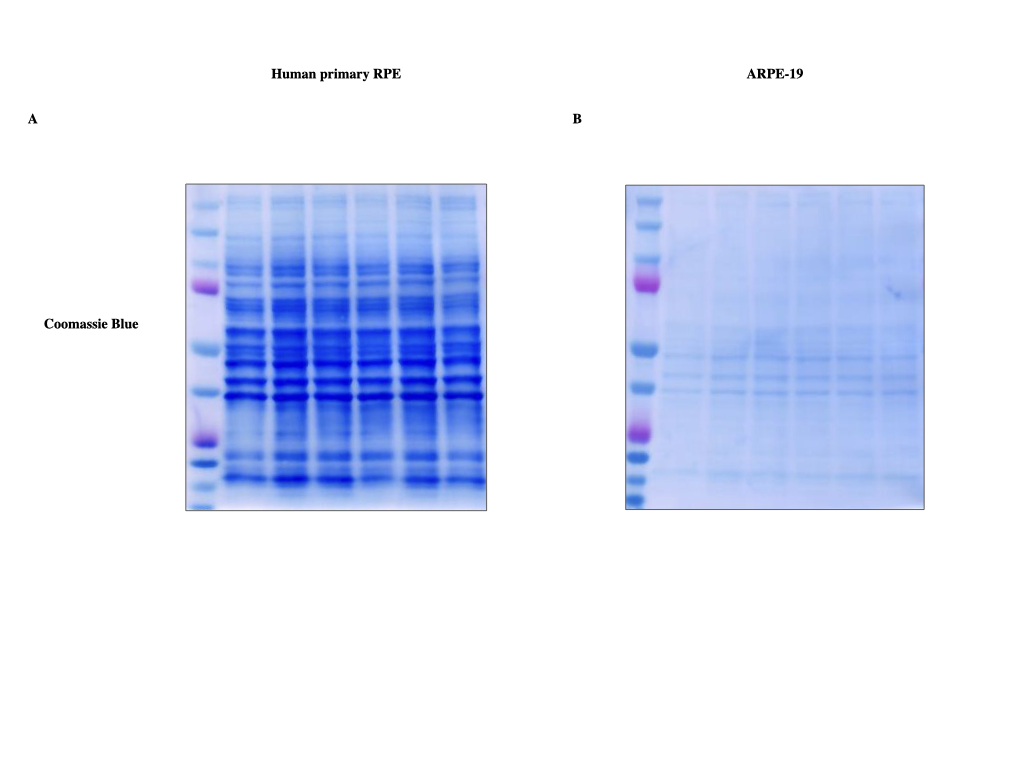

Supplement: S2 Fig — A. Whole blot image of the Coomassie staining corresponding to Fig 1A. B. Whole blot image of the Coomassie staining corresponding to Fig 1C. (TIFF) [file pone.0244307.s002.tiff]

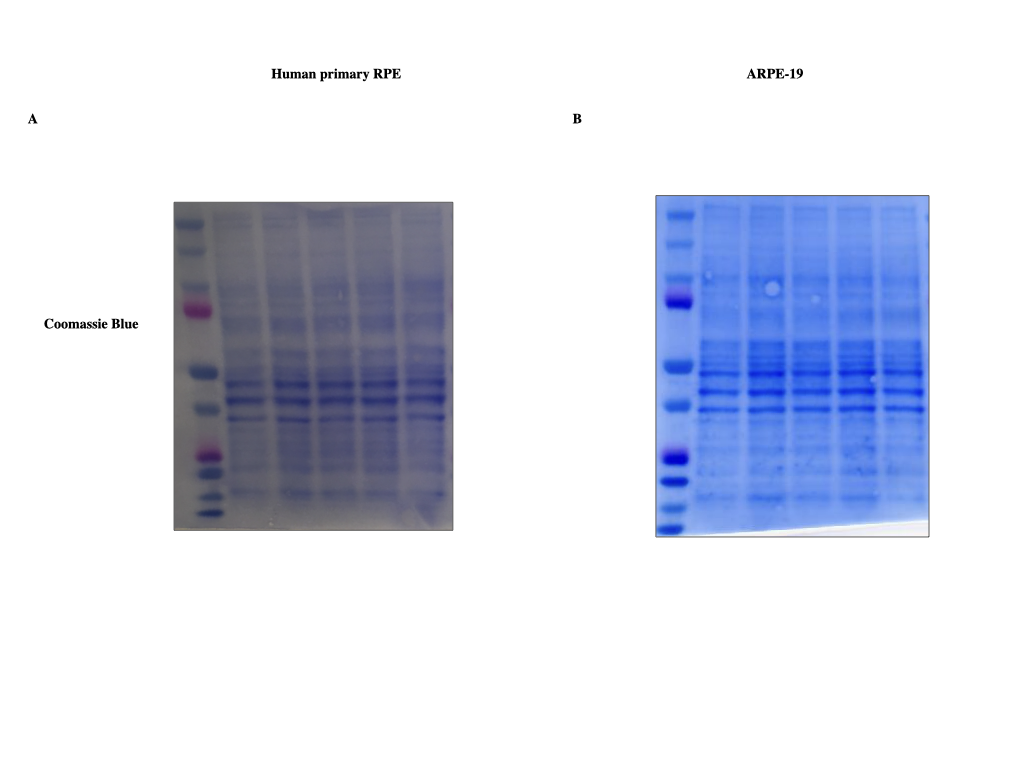

Supplement: S3 Fig — A. Whole blot image of the Coomassie staining corresponding to Fig 2A. B. Whole blot image of the Coomassie staining corresponding to Fig 2C. (TIFF) [file pone.0244307.s003.tiff]

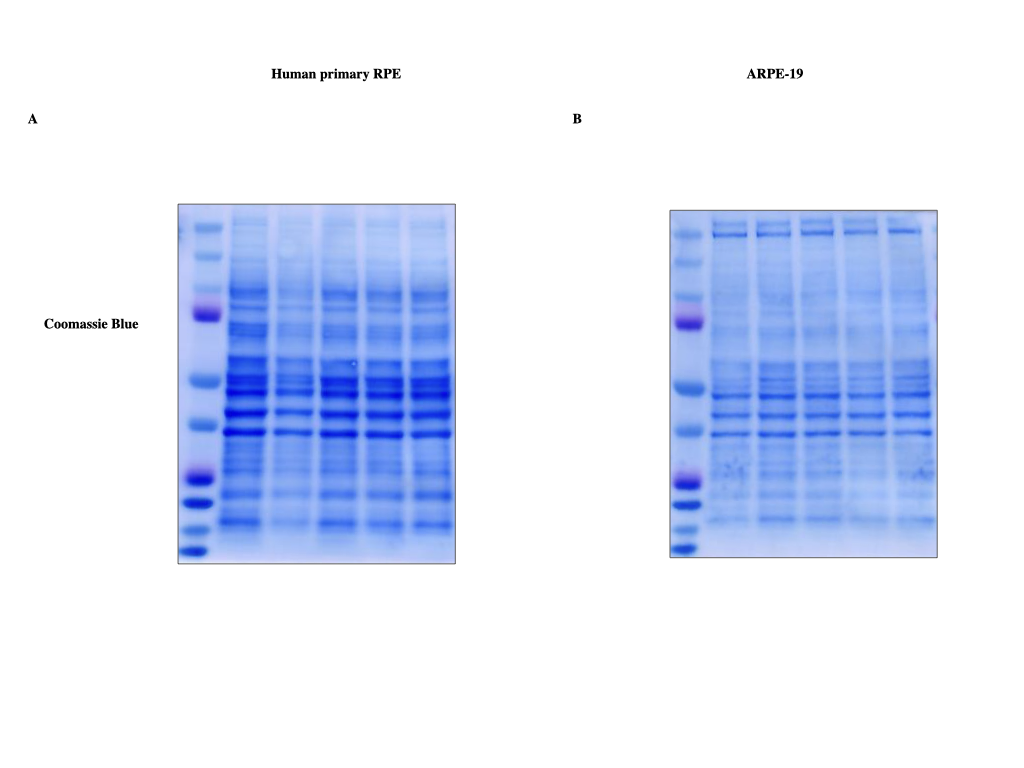

Supplement: S4 Fig — A. Whole blot image of the Coomassie staining corresponding to Fig 3A. B. Whole blot image of the Coomassie staining corresponding to Fig 3C. (TIFF) [file pone.0244307.s004.tiff]

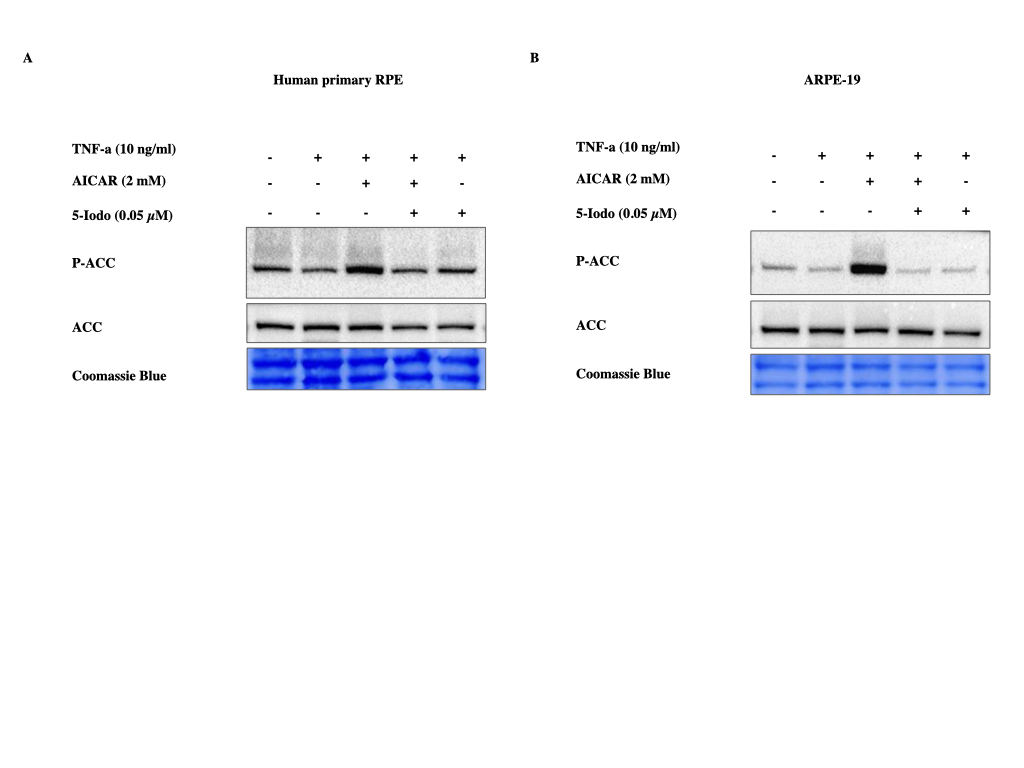

Supplement: S5 Fig — Acadesine induced AMPK activation (P-ACC) in the presence of TNF-a. Blockage of conversion of acadesine to ZMP by employment of 5-Iodo (0.05μM) prevented the AMPK activation (P-ACC) as seen in A. Western blot representing the AMPK activation (P-ACC) in human primary RPE lysates. B. Western blot representing the AMPK activation (P-ACC) in ARPE-19 cells lysates. (TIFF) [file pone.0244307.s005.tiff]

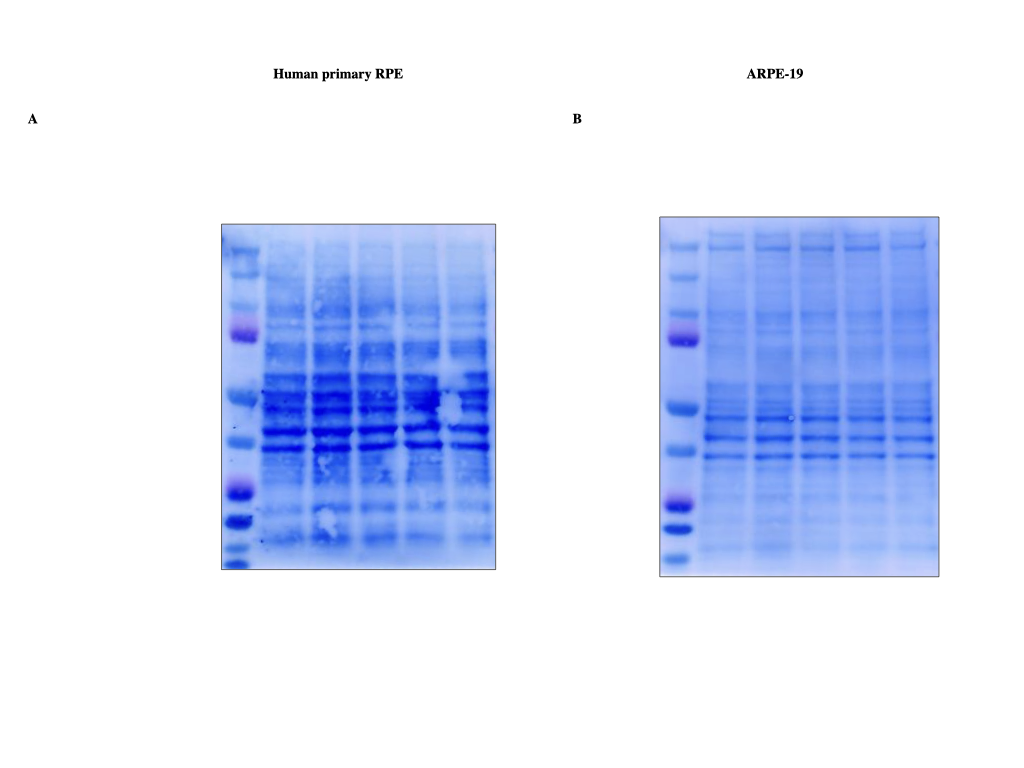

Supplement: S6 Fig — A. Whole blot image of the Coomassie staining corresponding to S5A Fig. B. Whole blot image of the Coomassie staining corresponding to S5B Fig. (TIFF) [file pone.0244307.s006.tiff]

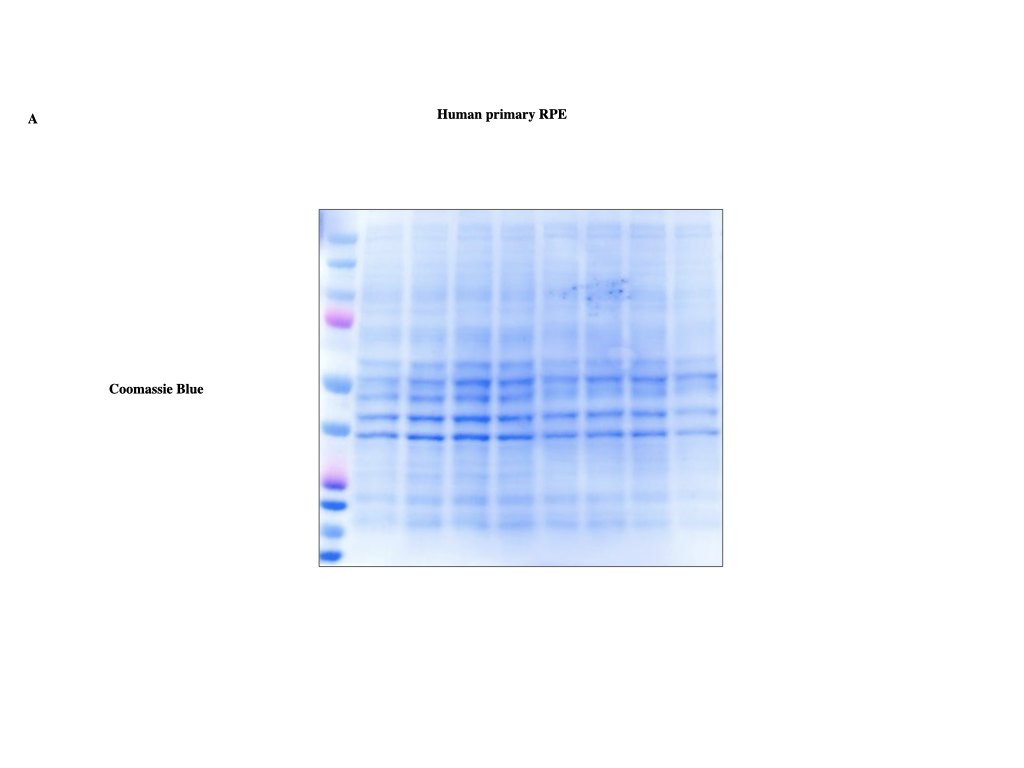

Supplement: S7 Fig — A. Whole blot image of the Coomassie staining corresponding to Fig 4A. (TIFF) [file pone.0244307.s007.tiff]

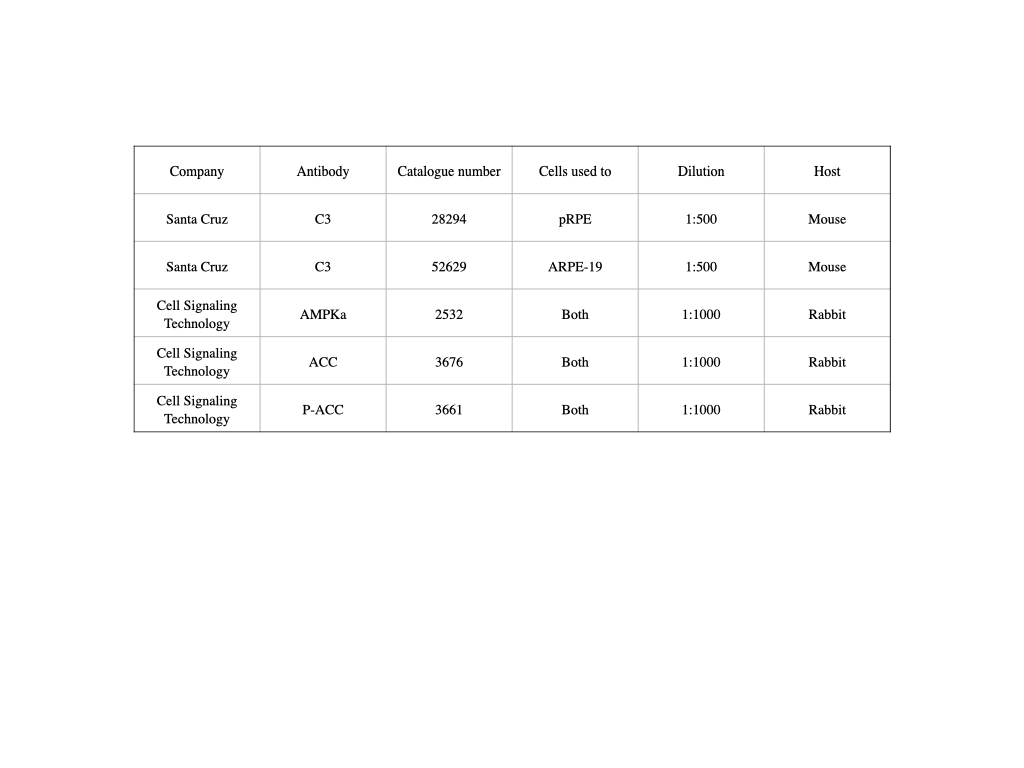

Supplement: S1 Table — (TIFF) [file pone.0244307.s008.tiff]
